# Two gap-free genomes of *Argentina* provide insights into their genetic relationships

**DOI:** 10.1186/s43897-025-00160-4

**Published:** 2025-08-04

**Authors:** Xien Wu, Qin Qiao, Qiang Cao, Zhongqiong Tian, La Qiong, Ticao Zhang

**Affiliations:** 1 State Key Laboratory of Phytochemistry and Natural Medicines, Kunming Institute of Botany, Chinese Academy of Sciences, Kunming, 650201 China; 2 College of Horticulture and Landscape, Yunnan Agricultural University, Kunming, 650201 China; 3 Key Laboratory of Biodiversity and Environment on the Qinghai-Tibetan Plateau, Ministry of Education, School of Ecology and Environment, Tibet University, Lhasa, 850000 China; 4 Yani Observation and Research Station for Wetland Ecosystem of the Tibet (Xizang) Autonomous Region, Tibet University, Lhasa, 850000 China

The Potentilleae of the Rosaceae family is one of the most diverse groups in the Northern Hemisphere, comprising approximately 13 genera and 1,700 species. It is a typical taxa representative of the varied ploidy levels within the Rosaceae, ranging from tetraploid (4x) to dodecaploid (12x), with diploids being relatively rare (Persson et al. 2020). Hybridization, accompanied by polyploidization, plays a critical role in generating the current taxonomic complexity within this group. Notably, *Argentina* represents the oldest lineage within Potentilleae, and is primarily distributed in the Tibetan Plateau (Xue et al. 2024). Tetraploid *Argentina anserina* (formerly *Potentilla anserina*) and diploid *Argentina lineata* (formerly *Potentilla fulgens*), are two closely related species widely distributed in the Tibetan Plateau, sharing similar morphological characteristics and overlapping distribution areas. *A. lineata* is utilized in traditional medicine by certain ethnic minorities in China and Northeast India for treating stomach ailments, diarrhea, oral ulcers, diabetes, etc.. Meanwhile, *A. anserina* serves not only as an important traditional medicine but also as a famous nutritional food for the Tibetan people, as it has enlarged tuberous roots containing a substantial amount of starch, polysaccharides, flavonoids and terpenes (Xu et al. 2010). Modern pharmacological research has shown that the polysaccharides in *A. anserina* possess antioxidant activity, aiding in the prevention and treatment of high-altitude pulmonary edema and cerebral edema (Shi et al. 2021). Although genomic data of *A. anserina* have been released (Gan et al. 2021), a high-quality gap-free genome assembly could provide deeper insights into the molecular basis of active compound biosynthesis and its evolutionary history.

To elucidate the origin, evolution, and genetic mechanisms underlying the unique characteristics of *A. anserina* and *A. lineata*, we generated chromosome-level genomes for both the tetraploid *A. anserina* and diploid *A. lineata* (Fig. 1A). Based on these reference genomes, we performed comparative genomics and phylogenetic analysis with other related species of Rosaceae to investigate their evolutionary history. Notably, our findings suggest that *A. lineata* is likely the diploid ancestral progenitor of *A. anserina*, providing novel insights into species divergence and the genetic evolution underlying local adaptation in both species.

**Fig. 1:**
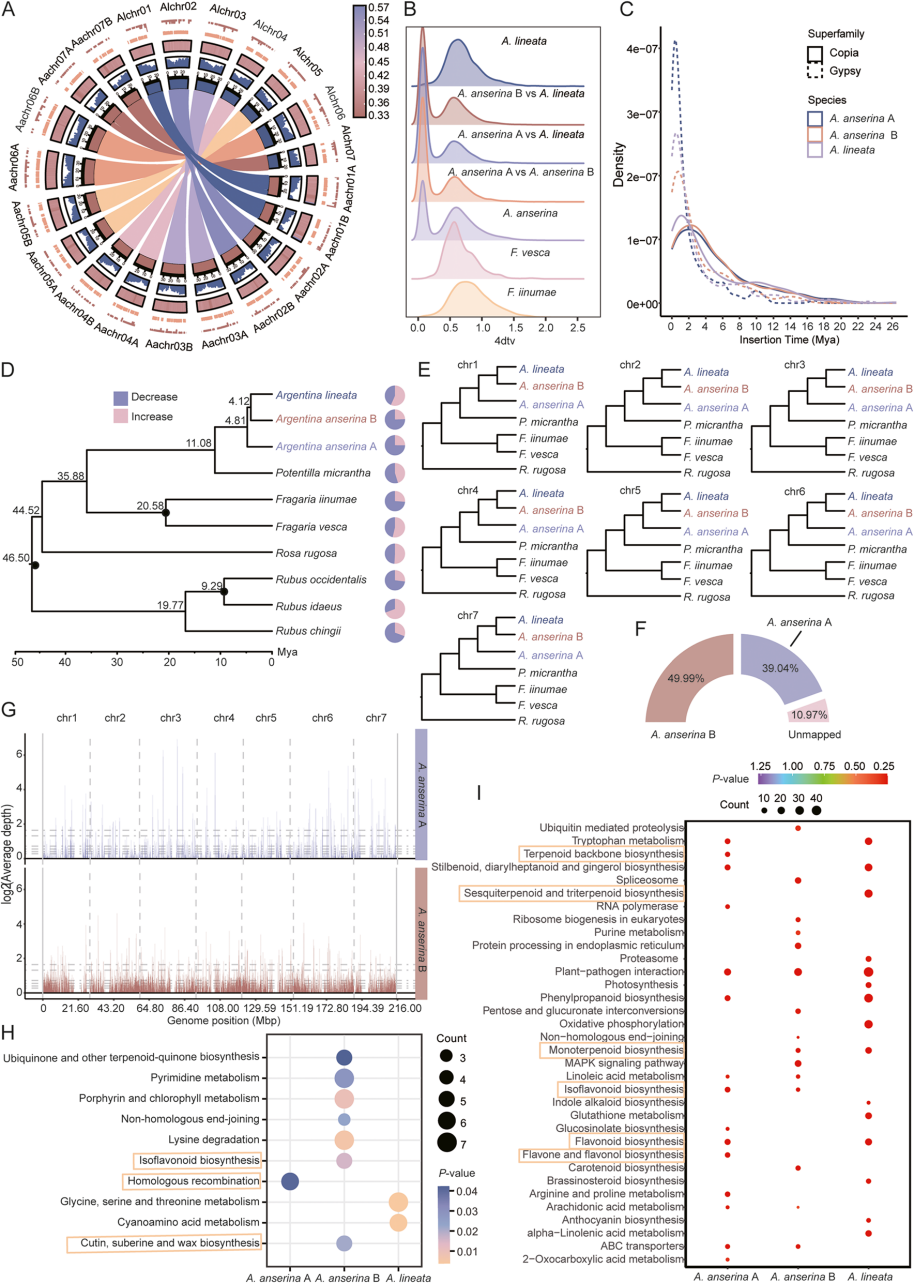
Comparative and evolutionary genomic analysis of *A. anserina* and *A. lineata*. **A** Genomic characteristics of *A. anserina* and *A. lineata*. Among them, Alchr01 - 07 is *A. lineata*, Aachr01 A- 07 A and Aachr01B- 07B are *A. anserina*. The circle diagram from outside to the inside is: Gypsy density, Copia density, GC content, gene density and collinear regions. **B** Four-fold degenerate synonymous sites of the third codon (4 dtv) analysis of *A. lineata*, *A. anserina*, *F. vesca* and *F. iinumae.* **C** Insertion times for Ty1/Copia, Ty3/Gypsy of *A. anserina* A, *A. anserina* B and *A. lineata*. **D** Phylogenetic tree of *A. lineata*, *A. anserina* and seven other species of the subfamily Rosoideae, with analyses of divergence times and gene family contraction and expansion. Species in red font are data from this study, black dots on the developmental tree are calibration points used to estimate divergence times, and numbers on the phylogenetic tree are estimated divergence times. Pie charts indicate the proportion of gene families that contracted and expanded. **E** A phylogenetic tree of the chromosomes of *A. anserina* A, *A. anserina* B and *A. lineata.* **F** Mapping quality of *A. lineata* in the composite reference genome (*A. anserina* A and *A. anserina* B). **G** Average depth of coverage of *A. lineata* in the composite reference genome (*A. anserina* A and *A. anserina* B). **H** KEGG enrichment analysis of positively selected genes. This figure shows the pathways that are significantly enriched (*P*-value < 0.05) and highlights some important pathways. **I** KEGG enrichment analysis of expansion gene. The figure shows significantly enriched pathways (*P*-value < 0.05) and highlights some important pathways

The *k*-mer analysis of genome survey indicated that the heterozygosity rate of *A. lineata* was approximately 0.3% (Figure S1 A), while that of *A. anserina* was about 0.6% (Figure S1B). Smudgeplot analysis of *k*-mer spectra showed a distribution with 47% *k*-mer pairs in an AABB ratio, suggesting that *A. anserina* is an allotetraploid (Figure S1D). We adopted Illumina short-read and PacBio HiFi long-read sequencing technologies to sequence the whole genomes of both species. For *A. lineata*, we generated 36.25 GB of Illumina data (~ 150 × coverage) and 24.93 GB of HiFi data (~ 100 × coverage). For *A. anserina*, we obtained 72.53 GB of Illumina data (~ 150 × coverage) and 35.73 GB of HiFi data (~ 90 × coverage) (Table S1). The genome assembly of *A. lineata* resulted in a genome size of 187.88 Mb with a contig N50 of 26.06 Mb. Utilizing 32.58 GB of Hi-C sequencing data, we successfully anchored the chromosome-level genome of *A. lineata* to seven pseudo-chromosomes (Figure S1E). The final assembled genome size for *A. anserina* is 431.96 Mb, with a contig N50 of 30.08 Mb. Based on 47.64 GB of Hi-C sequencing data, we successfully anchored the genome of *A. anserina* to 14 pseudo-chromosomes, which can be divided into subgenomes A and B (Figure S1 F). These efforts resulted in gap-free chromosome-level genome assemblies for *A. lineata* and *A. anserina*, representing the highest quality genome assemblies.

Benchmarking Universal Single-Copy Orthologs (BUSCO) assessment of the two genomes indicated that the intact core genes of *A. lineata* and *A. anserina* were 94.6% and 95.7%, respectively, indicating high genome integrity (Table S2). We annotated the genomes of both species and predicted 24,261 and 43,542 protein-coding genes in the genomes of *A. lineata* and *A. anserina*, respectively. Gene annotations were performed against GO, KEGG, SwissProt databases, with 97.64% of *A. lineata* genes and 98.28% of *A. anserina* genes annotated in at least one database (Table S3).

We conducted a collinearity analysis on subgenomes A and B of *A. anserina* as well as *A. lineata* using *A. lineata* as the reference genome. *A. lineata* exhibited a high level of synteny with both subgenomes A and B of *A. anserina* (Figure S2). We also performed a comparative genomic analysis between our newly assembled genome and the published *A. anserina* genome (Gan et al. 2021). The results demonstrated strong collinearity between the two genomes (Figure S3). We also calculated the four-fold degenerate synonymous sites of the third codon (4 dtv) rate to identify putative whole genome duplication (WGD) events in *A. anserina* and *A. lineata* (Fig. 1B). The results revealed that all four species shared one peak around 0.6 corresponding to the paleo-hexaploidization event common to core eudicots. Whereas, *A. anserina* exhibited an additional prominent peak around 0.1, similar to previously reported results for *A. anserina* (Ks∼0.08) (Gan et al. 2021), further supporting a recent tetraploidization event in *A. anserina*.

The subgenomes A (216.02 Mbp) and B (215.41 Mbp) of *A. anserina* are larger than the genome of *A. lineata* (187.88 Mbp), possibly due to increased transposable element (TE) content. TEs contribute approximately 38.67% and 39.70% of the sequences in the A and B subgenomes of *A. anserina*, respectively, while they contribute 29.19% in *A. lineata* (Table S4). Among these, long terminal repeat retrotransposons (LTR-RTs) are the most abundant TEs, particularly the Ty1/Copia and Ty3/Gypsy superfamilies (Figure S4 A). The Ty1/Copia family, Ale is the most abundant (Figure S4B), while Tekay dominates within the Ty3/Gypsy family (Figure S4 C). To further understand the dynamics in LTR-RTs during the evolutionary process, we estimated their insertion times. The results indicated that the insertion times of LTR-RTs were largely synchronous between subgenomes A and B of *A. anserina*, indicating that the proliferation of LTR-RTs likely occurred subsequent to the tetraploidization event in *A. anserina*. Furthermore, Gypsy-LTR-RTs showed relatively recent insertions (~ 1 Mya) compared to Copia-LTR-RTs (~ 2 Mya) across all three (sub)genomes (Fig. 1C), suggesting ongoing retrotransposon activity. Similar insertions of LTR-RTs have also been observed in other plants on the Tibetan Plateau (Zhang et al. 2019), which may be related to the harsh environmental conditions caused by the rapid uplift of the plateau.

To estimate the degree of evolutionary relatedness of *A. anserina* and *A. lineata*, we first constructed a phylogenomic tree for 16 species from 11 genera within the Rosaceae, along with three outgroups, based on one-by-one orthologs (Table S5). The results indicated that *A. anserina* and *A. lineata* formed a monophyletic clade, suggesting a closer evolutionary relationship between them (Figure S5). To further investigate the evolutionary history of *A. anserina* and *A. lineata*, we also constructed phylogenetic trees for 25 related species from Potentilleae, using nuclear (ITS and ETS) and chloroplast (*trnL-trnF* and *trnG-trnS*) sequences, respectively (Table S6). Both trees resolved *Potentilla* and *Argentina* as two well-supported clades*.* Within the clade of *Argentina*, the nuclear tree showed that *A. anserina, Argentina microphylla* and *A. lineata* grouped in a single cluster (Figure S6 A). While the chloroplast tree indicated a more distant relationship between *A. anserina* and *A. lineata* (Figure S6B). The inconsistency between nuclear and chloroplast phylogenies has been observed in other studies, which could be caused by hybridization or incomplete lineage sorting (ILS) events in history (Xue et al. 2024). Regardless, both trees constructed from the nuclear genome and nuclear genes indicated a close relationship between *A. anserina* and *A. lineata*.

Potentilleae exhibits mixed ploidy levels, ranging from tetraploid (4x) to dodecaploid (12x), with diploids being rare. Hybridization accompanied by genome polyploidy is considered one of the important evolutionary processes in Potentilleae, contributing to the scarcity of diploid species (Persson et al. 2020). Studies suggest that Potentilleae originated in the Tibetan Plateau, where *Argentina* is widely distributed in high-altitude regions, especially in the Hengduan Mountains. As elevation increases and temperatures decrease, *Argentina* has undergone polyploidization and developed certain traits to adapt to extreme environments (Xue et al. 2024). In this study, we investigated the allotetraploid *A. anserina* and the diploid *A. lineata*, which share highly overlapping geographic distributions in southwestern China and the Tibetan Plateau. In addition, there are numerous morphological similarities between *A. anserina* and *A. lineata*. Both species are perennial herbs with similar radical and stem leaves, interrupted pinnate leaf blades (usually elliptic), dense white tomentum on the leaf underside,, and yellow, obovate petals with rounded apices. Based on these observations and phylogenetic evidence, we speculate that *A. lineata* may be one of the diploid progenitors of *A. anserina*.

To test this hypothesis, we constructed a phylogenetic tree based on whole-genome data and estimated the divergence time of *A. lineata* and the subgenomes of *A. anserina*. Subgenome B of *A. anserina* clusters with *A. lineata* first, with an estimated divergence time of about 4.12 Mya, whereas subgenome A of *A. anserina* diverged earlier, around 4.81 Mya (Fig. 1D). Next, we constructed phylogenetic trees using seven chromosomes respectively. Notably, phylogenetic analysis revealed that all seven chromosomes of subgenome B of *A. anserina* are closely related to *A. lineata* (Fig. 1E)*.* This result further supports the allopolyploid origin of *A. anserina* and suggests *A. lineata* likely to be a potential diploid progenitor.

Given the limitation of phylogenetic methods, we also employed sppIDer analysis to map short-read sequencing data of *A. lineata* to a composite reference genome constructed from subgenomes A and B of *A. anserina*. Mapping quality (MQ) results showed that 89.03% of reads from *A. lineata* were successfully mapped to the reference genome, with 39.04% mapped to subgenome A and 49.99% mapped to subgenome B of *A. anserina* (Fig. 1F). The average coverage depth of *A. lineata* in subgenome A was 33.46, compared to 45.36 in subgenome B, suggesting that *A. lineata* shares more sequence similarities with subgenome B of *A. anserina*. Analysis of coverage bins for *A. lineata* showed that 9.95% of the bins in subgenome A are above the minimum coverage threshold, while 41.55% of the bins in subgenome B exceed this threshold (Fig. 1G and Table S7). Additionally, the proportion of bins in subgenome B is generally higher than that in subgenome A across all coverage bins, especially in bin 4 (65.05 ×—98.46 ×) (Fig. 1G and Table S7). These results suggest that more than half of the bins in *A. anserina* may originate from *A. lineata*. Compared with subgenome A, *A. lineata* has made a more significant contribution to the genome composition of subgenome B in *A. anserina*. In addition, the average coverage in some regions is extremely high, which may be caused by repetitive sequences. Therefore, multiple lines of evidences support our speculation that *A. lineata* may be a potential ancestral diploid of *A. anserina*. However, the limited availability of genomic data for Potentilleae species necessitates further validation of this hypothesis with additional data.

The genus *Argentina* is widely distributed in high-altitude areas, especially in the Hengduan Mountains. As the altitude increases, plants will face environmental stresses such as low temperature, low oxygen, and strong ultraviolet radiation. Therefore, their genes will experience different selection pressures and polyploidization processes, and eventually develop some adaptive traits to cope with the challenges of extreme environments (Xue et al. 2024). To investigate the genetic basis of these adaptations, we conducted a positive selection analysis on *A. anserina* and *A. lineata*. The results showed that 880, 1152 and 746 genes were under significant positive selection in *A. anserina* A, *A. anserina* B, and *A. lineata*, respectively (Table S8). The pathways significantly enriched in *A. lineata* are cyanoamino acid metabolism and glycine, serine and threonine metabolism. Compared with *A. lineata*, the pathways significantly enriched by the positively selected genes in *A. anserina* are highly relevant to the adaptation to the extreme environment of the Tibetan Plateau. The pathway significantly enriched in *A. anserina* A is homologous recombination (HR). HR repair is one of the highly efficient defense mechanisms evolved by plants to cope with extreme environments and can repair DNA damage precisely. The pathways significantly enriched by the positively selected genes in *A. anserina* B include cutin, suberin and wax biosynthesis, isoflavonoid biosynthesis, etc. (Fig. 1H and Table S9). Among them, the cuticle, as a protective shield covering the outer walls of epidermal cells of plant organs, has made great contributions to the adaptation of plants to environmental stresses.

Moreover, we conducted gene family clustering analysis on *A. lineata*, *A. anserina*, and seven other species from the Rosoideae subfamilies using CAFE (v.4.2.1). A total of 28,304 orthogroups were identified, with 11,023 orthogroups shared among all species. Among these, 5,592 were single-copy gene families. *A. lineata*, subgenomes A and B of *A. anserina* have 89, 36, and 49 unique gene families, respectively (Figure S7 A). Interestingly, *A. lineata* exhibited a higher number of expanded gene families, whereas subgenomes of *A. anserina* showed a predominance of contracted gene families. This may be related to the WGD event in *A. anserina*. Studies have found that WGD is often followed by gene loss and diploidization, which may increase the rate of gene gain and loss (Guo 2012). In addition, during the evolution of polyploid species, genes may be selectively retained or lost, with some genes related to environmental adaptation being preferentially retained (Chen et al. 2020).

Then, we performed KEGG enrichment analysis on these contracted and expanded gene families (Table S10, Figure S7B and Fig. 1I). In *A. lineata*, the expanded genes were significantly enriched in pathways related to sesquiterpenoid and triterpenoid biosynthesis, as well as monoterpenoid biosynthesis. The genes significantly enriched in sesquiterpenoid and triterpenoid biosynthesis pathway (*P*-value = 5.08E- 15) were annotated as (–)‑germacrene D synthase (*GERD*), which belong to the TPS-a subfamily. Among them, the genes *GERD* (*Poful02G0095700*, *Poful03G0182100*) were undergoing expansion while being under positive selection (Table S8). The TPS family members are crucial for the diversity of bioactive terpenoid compounds in plants, and their lineage-specific expansion contributes to the modular metabolic network that maximizes the production of related compounds (Karunanithi and Zerbe 2019). Terpenoids play essential roles in plant growth, development, defense, and environmental adaptation. Moreover, terpenoid compounds are also closely related to the medicinal value of plants. Research has found that triterpenoid compounds extracted from *A. lineata* exhibit inhibitory activity against α-glucosidase, which is helpful for the treatment of diabetes (Anshika et al. 2022).

In *A. anserina*, the expanded genes in both subgenomes A and B were enriched in pathways related to flavonoid and terpenoid synthesis, such as flavone and flavonol biosynthesis, isoflavonoid biosynthesis and monoterpenoid biosynthesis. These compounds are significant for the medicinal value and stress resistance of plants. Especially flavonoids, which can accumulate in plant epidermal cells as sunscreen to avoid UV-B damage, and may contribute to the adaptation of *A. anserina* to the environment with intense ultraviolet radiation on the Tibetan Plateau. Notably, the expanded genes in *A. anserina* were enriched in more flavonoid-related pathways compared to *A. lineata*. In the subgenome A of *A. anserina,* the genes related to flavonoid synthesis were annotated as isoflavone 7-O-glucoside- 6-O-malonyltransferase (*IF7MaT*) and shikimate O-hydroxycinnamoyl transferase (*HCT*). In the subgenome B of *A. anserina*, the genes related to flavonoid synthesis were annotated as isoflavone/4'-methoxyisoflavone 2'-hydroxylase (*CYP81E*), among which the gene *CYP81E* (*Poans01bG0135300*) was also under positive selection (Table S8). Studies have shown that plants can adapt to different light intensities by regulating the activities of these genes (Procko et al. 2022). Therefore, compared with *A. lineata* (a.s.l. 1100 - 3600 m), *A. anserina* may be better adapted to extreme environments, contributing to its higher altitude habitats and wider distribution range (a.s.l. 600–4100 m).

In conclusion, this study presents the gap-free genomes of the allotetraploid *A. anserina* and diploid *A. lineata*, both distributed on the Tibetan Plateau and possessing significant values in traditional medicine and nutritional food. Phylogenomic analyses revealed a close relationship between *A. lineata* and subgenome B of *A. anserina*, suggesting that *A. lineata* may represent a potential ancestral diploid of *A. anserina*. This study identified a significant role of transposable elements, particularly LTR-RTs, in genome size expansion following polyploidization events. Comparative genomic analysis also indicates that in *A. anserina*, significantly positively selected genes are more enriched in pathways related to adapting to the extreme environment of the Tibetan Plateau. In addition, expanded genes in *A. lineata* are mainly associated with its medicinal value, while expanded genes in *A. anserina* are related to both medicinal value and stress resistance. Our findings provide valuable insights into species divergence and genome evolution in high-altitude environments.

## Supplementary Information

Supplementary Material 1.

Supplementary Material 2.

## Data Availability

The raw genomic reads generated in this study have been deposited in the NCBI Sequence Read Archive (BioProject PRJNA1197098). The genome assembly and annotation files generated in this study can be obtained from the China National Center for Bioinformation (BioProject PRJCA033495) and the Genome Database for Rosaceae (https://www.rosaceae.org/).
