# Supplementary material for: Two gap-free genomes of Argentina provide insights into their genetic relationships
Source: Mol Hortic. 2025 Aug 4;5:50. doi: 10.1186/s43897-025-00160-4 (PMC12320355; doi:10.1186/s43897-025-00160-4)
Supplement: Supplementary file 1 — Supplementary Material 1. [file 43897_2025_160_MOESM1_ESM.docx]

**Two gap-free genomes of *Argentina* provide insights into their genetic relationships**

**Xien Wu ^1#^, Qin Qiao ^2#^, Qiang Cao ^2^, Zhongqiong Tian ^3^, La Qiong ^3,4*^, Ticao Zhang ^1*^**

^1^ State Key Laboratory of Phytochemistry and Natural Medicines, Kunming Institute of Botany, Chinese Academy of Sciences, Kunming 650201, China

^2^ College of Horticulture and Landscape, Yunnan Agricultural University, Kunming 650201, China

^3^ Key Laboratory of Biodiversity and Environment on the Qinghai-Tibetan Plateau, Ministry of Education, School of Ecology and Environment, Tibet University, Lhasa 850000, China

^4^ Yani Observation and Research Station for Wetland Ecosystem of the Tibet (Xizang) Autonomous Region, Tibet University, Lhasa 850000, China

^#^ The authors contribute equally to this work.

* Author for correspondence: lhagchong@163.com; [zhangticao@mail.kib.ac.cn](mailto:zhangticao@mail.kib.ac.cn).

**Materials and methods**

**Plant materials**

Young leaves of *Argentina anserina* from Mozhugongka, Lhasa, Tibet (91°45′E, 29°50′N, altitude: 4,342 m) and *Argentina lineata* from Liangwang Mountain, Kunming, Yunnan (102°70′E, 24°73′N, altitude: 2,820 m) were collected for whole genome sequencing. The collected leaves were immediately frozen in liquid nitrogen for subsequent genomic DNA extraction.

**Genome sequencing**

The qualified DNA samples were fragmented, purified, repaired, and ligated with sequencing adapters. Libraries were constructed from the processed DNA samples and sequenced using the Illumina HiSeq platform. The raw data obtained from sequencing were filtered to remove low-quality reads. *k*-mer (k=17) analysis was performed based on the reads after quality control to estimate the genome size, heterozygosity, duplicity. In addition, high quality sample DNA was processed and the constructed library was loaded into the PacBio Sequel II platform for sequencing; the raw data was processed using the ccs program (https://github.com/PacificBiosciences/ccs) to generate HiFi reads. In order to obtain genomes at the chromosome level, we performed Hi-C library construction and sequencing of *A. anserina* and *A. lineata*. The samples were first cross-linked and fixed and cell lysed, the DNA was processed using restriction endonucleases, then end repairs were performed while adding biotin, adjacent DNA fragments were ligated, de-crosslinked, fragmented again, and finally the biotin-containing fragments were captured to construct the libraries and sequenced.

**Genome assembly and annotation**

We used hifiasm (version 0.16.1-r375) (Cheng et al. 2021) for the initial assembly of PacBio HiFi reads, followed by Juicer (Durand et al. 2016) to align Hi-C reads to the preliminarily assembled genome, preliminary Hi-C-assisted chromosome assembly by 3D-DNA (Dudchenko et al. 2017), and hand-checked adjustments using Juicebox (Robinson et al. 2018). Based on HiFi reads the gap was filled with LR_Gapcloser software (Xu et al. 2019). Most chromosomes have assembled the telomere characteristic sequence (TTTAGGG)n at their telomere ends. For chromosomes with short or missing telomere characteristic sequence, we re-align HiFi reads to the chromosomes, extract reads near the telomeres, assemble them into contigs using hifiasm, then align the contig with the chromosome and extend the chromosome outward to assemble the telomere sequence as completely as possible. Through the identification of the distribution of characteristic sequences on the chromosomes of *A. anserina* (Figure S8), it was found that most chromosomes contain telomere characteristic sequences. Meanwhile, a highly tandem repetitive sequence was identified and presumed to be the centromere, with its unit sequence being AAAGATACTCAGTTTGAAAGAAACACACTCCCACACTTGCAAAACTCTCAAATTCAATAGAAACTCGCATCAGAAAGCCTTACAGAAAGATACTCAAACGAGAAATGAAAAGATACACAATTGGAATAGTAAAGATACGCAAACGATGT. In addition, the 18-5.8-28S rDNA array is distributed on multiple chromosomes, and the 5S rDNA array is located on only one chromosome. Regarding the identification of the distribution of characteristic sequences on the chromosomes of *A. lineata* (Figure S9), the results show that the chromosomes generally contain telomere characteristic sequences. We also identified a highly tandem repetitive sequence which is presumed to be the centromere, and its unit sequence is AGGTACATTTTATTCCCTTCCGGTAACATCACAAATATATGCACTTCGACTATACAAATATTCCATCCAAAATCCTCCAATTTACCGAG. The 18 - 5.8 - 28S rDNA array is distributed on one chromosome, and the 5S rDNA array is distributed on two chromosomes. Chloroplast and mitochondrial genomes were assembled separately using GetOrganelle (Jin et al. 2020). The genome was polished in two rounds with NextPolish (Hu et al. 2020), and Redundans (Pryszcz and Gabaldón 2016) were used to identify redundancies in the scattered sequences, as well as to remove redundancies and exogenous contamination. To assess the quality of genome assembly, next-generation reads, third-generation reads, and RNA-Seq reads were mapped to the genome using BWA (Li 2013), Minimap2 (Li 2018), and hisat2 (Kim et al. 2015), respectively. Finally, the completeness of the genome was assessed using BUSCO (Simão et al. 2015).

Methods for gene structure prediction include (1) homology prediction: The genome sequences of *A. anserina* and *A. lineata* were aligned with 333,558 non-redundant protein sequences from *Potentilla micrantha*, *Potentilla anserina*, *Fragaria vesca*, *Rosa chinensis*, *Rubus chingii*, *Prunus persica*, *Malus domestica*, *Gillenia trifoliata*, *Dryas drummondii*, *Morus notabilis*, *Cannabis sativa*, *Ziziphus jujuba*, *Vitis vinifera*, and *Arabidopsis thaliana* to provide homologous protein evidence. (2) Transcript prediction: first, prepare the transcripts using next-generation sequencing data. Specific steps include de novo assembly using Trinity (Grabherr et al. 2011), and alignment of reads to the genome using hisat2, followed by transcript assembly using StringTie (Pertea et al. 2015). All transcript sequences were then merged and redundancy was removed using CD-HIT (Fu et al. 2012). Based on transcript evidence, gene structures were annotated using the PASA process (Haas et al. 2003) and full-length genes were identified by alignment with reference proteins, and finally AUGUSTUS (Stanke et al. 2008) was trained and optimised based on the full-length genes. (3) De novo prediction: finally, the MAKER2 (Cantarel et al. 2008) annotation process was used to combine the results of de novo prediction, transcript evidence and homologous protein alignment for comprehensive annotation, and EVidenceModeler (Haas et al. 2008) was used to integrate the annotation results from MAKER2 and PASA to generate consistent and high-quality gene annotation results.

Annotation of non-coding RNAs: tRNAs were annotated using tRNAScan-SE (Lowe and Eddy 1997). rRNAs were annotated using barrnap (https://github.com/tseemann/barrnap). other types of non-coding RNAs were annotated using RfamScan (Nawrocki et al. 2015).

The methods of gene function annotation include: using eggNOG-mapper (Huerta-Cepas et al. 2017) annotation to align the sequence with the eggNOG database to annotate the function of the gene; using DIAMOND (Buchfink et al. 2015) to align the protein sequence with the protein database; and using InterProScan (Jones et al. 2014) to align the sequences in InterPro's database to obtain the conserved sequences of the genes.

We used EDTA (Ou et al. 2019) to initially annotate the Transposable elements of *A. anserina* and *A. lineata*, and DeepTE (Yan et al. 2020) was used to optimise and re-annotate the sequences that were not annotated to the LTR-RTs. The annotated LTR-RTs were then further classified using TEsorter (Zhang et al. 2022). Multiple sequence comparison of LTR-RTs protein sequences was performed using MAFFT (Katoh and Standley 2013), and IQ-TREE 2 (Minh et al. 2020) was used to construct a phylogenetic tree of LTR-RTs.

**Comparative genome analysis**

We used TBtools (Chengjie et al. 2020) to draw the circular genome map and applied OrthoIndex (Zhang et al. 2024) to filter the collinearity relationship between *A. anserina* and *A. lineata*. Download the genome sequences, coding region sequences, protein sequences and annotation files of the 19 species that have been published; species details are given in Supplementary Table 4, and clustering analyses of gene families were performed for these species using OrthoFinder (Emms and Kelly 2019). The species tree multi-sequence alignment file generated by OrthoFinder is filtered using trimAl (Capella-Gutiérrez et al. 2009), and then the phylogenetic tree of the species is constructed using RAxML (Stamatakis 2014) software with the PROTGAMMAJTT model. The divergence times of the species were estimated using MCMCTree (Yang 2007) based on the results of the phylogenetic tree, where information on time-corrected points was obtained from the TimeTree website (Kumar et al. 2022).

We used the codeml program in the PAML software (Yang 2007) package to conduct positive selection analysis, with *A. anserina* A, *A. anserina* B, and *A. lineata* as the foreground branches, respectively. *F. iinumae*, *F. vesca*, *P. micrantha*, and *R. rugosa* as the background branches. The branch-site model was applied to analyze single-copy orthologous genes across these species. Then, the likelihood ratio test (LRT) was employed to compare the goodness of fit between the alternative model and the null hypothesis model. By calculating the difference in twice the log-likelihood and comparing it with the chi-square distribution, the P-values were obtained. Meanwhile, the false discovery rate (FDR) correction was performed on the *P*-values. Finally, the bayes empirical bayes (BEB) method was used to detect the positively selected sites within the genes. Based on the criteria of ω (Ka/Ks) > 1, *P*-values < 0.01 and FDR < 0.05, combined with the results of BEB, the genes under significant positive selection were identified. According to the results of OrthoFinder gene family clustering and species evolutionary tree, we used CAFÉ software (Mendes et al. 2020) to conduct evolutionary analysis of gene families on species tree to predict the contraction and expansion of species gene families, and then used the clusterProfiler package (Wu et al. 2021) to perform KEGG enrichment analysis on genes that contracted and expanded.

BLAST was used to compare the protein sequences of *A. anserina* and *A. lineata*, and to extract collinear regions between species. The location information of the genes was extracted from the annotation file, and the collinearity of these regions was analyzed using MCScanX (Wang et al. 2012) Synteny and rearrangement plot using SyRI (Goel et al. 2019). ParaAT (Zhang et al. 2012) was used to compare collinear gene pairs of *A. anserina*, *A. lineata*, *Fragaria iinumae* and *Fragaria vesca*, then ka, ks, ka/ks and 4DTv distance were calculated, and the distribution map of 4dtv was drawn.

A composite reference genome was generated from the genomes of *A. lineata* and *Potentilla micrantha* by using sppIDer (Langdon et al. 2018), and then the Illumina sequencing data of *A. lineata* was mapped to the composite reference genome. Four gene fragments (ITS, ETS, *trnL-trnF*, and *trnS-trnG*) were downloaded from NCBI for the 25 species (Feng et al. 2017) of the Potentilleae and the outgroups *Rosa majalis* and *Sanguisorba officinalis* (Table S5). These fragments were aligned to the genomes and chloroplast genomes of *A. lineata* and *A. anserina* using BLAST, and then the fragments with the highest BLAST scores were extracted using BEDTools (Quinlan and Hall 2010). Multiple sequence alignment of nuclear (ETS+ITS) and plastid (*trnL-trnF* + *trnS-trnG*) sequences was performed using MAFFT, nuclear and plastid gene fragments phylogenetic trees were constructed using IQ-TREE 2.


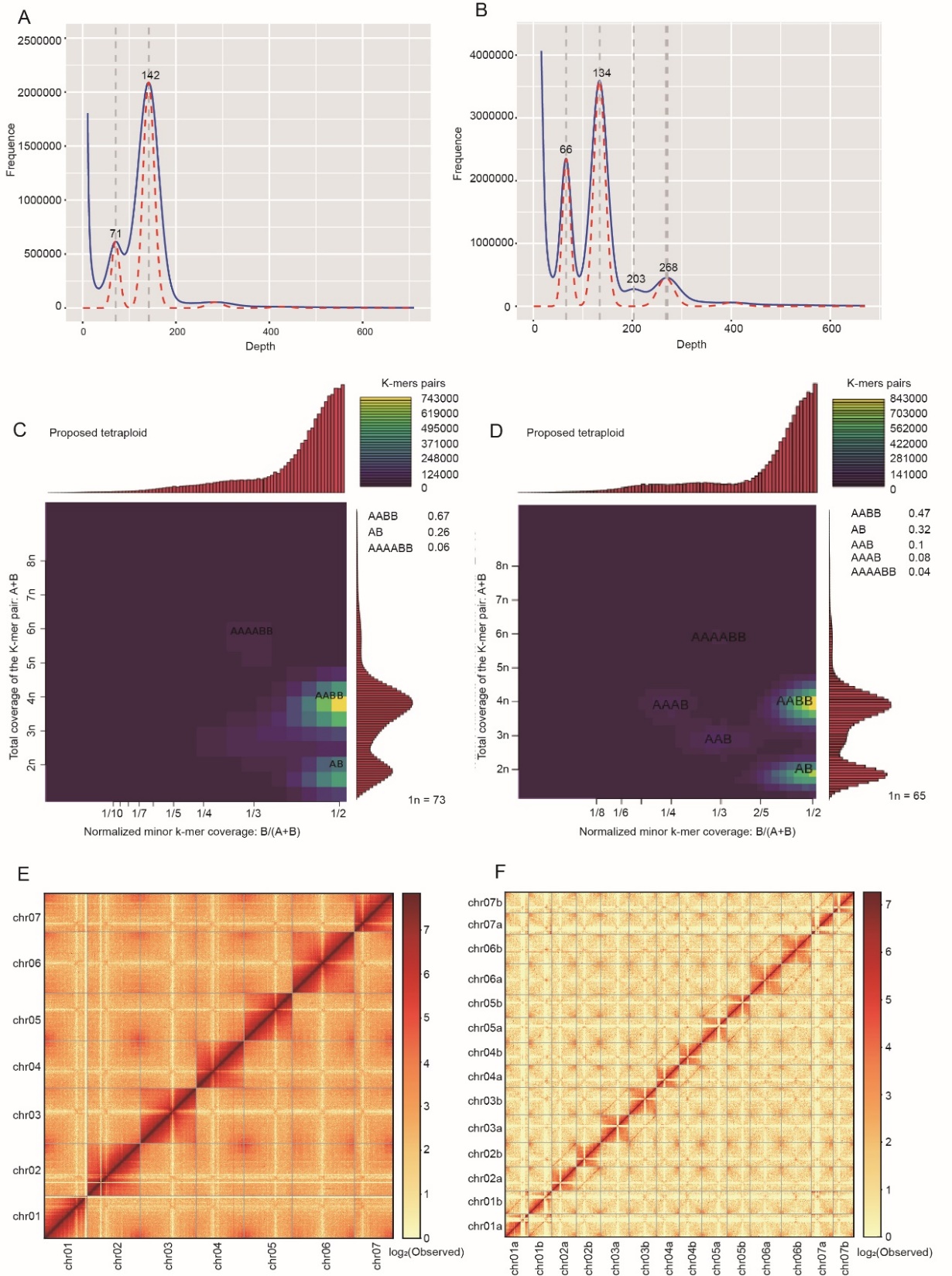


**Supplemental Figure S1** *K*-mer frequency distribution, smudgeplot analysis and Hi-C interaction heatmap. (A) *K*-mer frequency distribution of *A. lineata.* (B) *K*-mer frequency distribution of *A. anserina.* (C) Smudgeplot results for *A. lineata*. The speculated tetraploid may be due to bias (when the genome is low heterozygous, *k*-mer pairs only account for a small portion and cannot represent the wholegenome data). (D) Smudgeplot results for *A. anserina*. (E) Hi-C interaction heatmap of *A. lineata.* (F) Hi-C interaction heatmap of *A. anserina.*


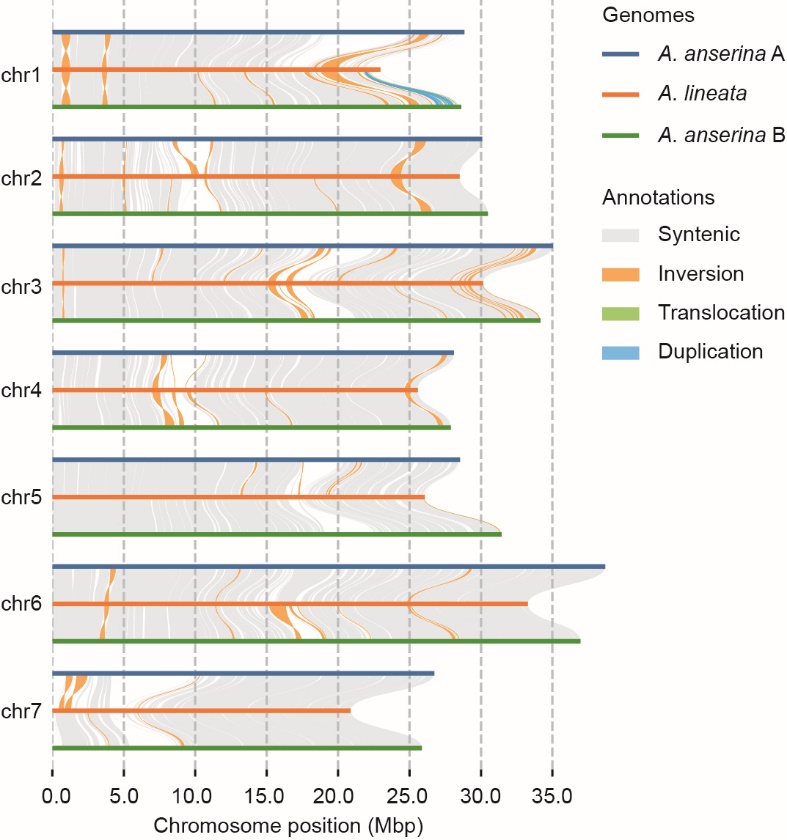


**Supplemental Figure S2** Synteny and rearrangement plot of *A. lineata*, *A. anserina* A and *A. anserina* B.


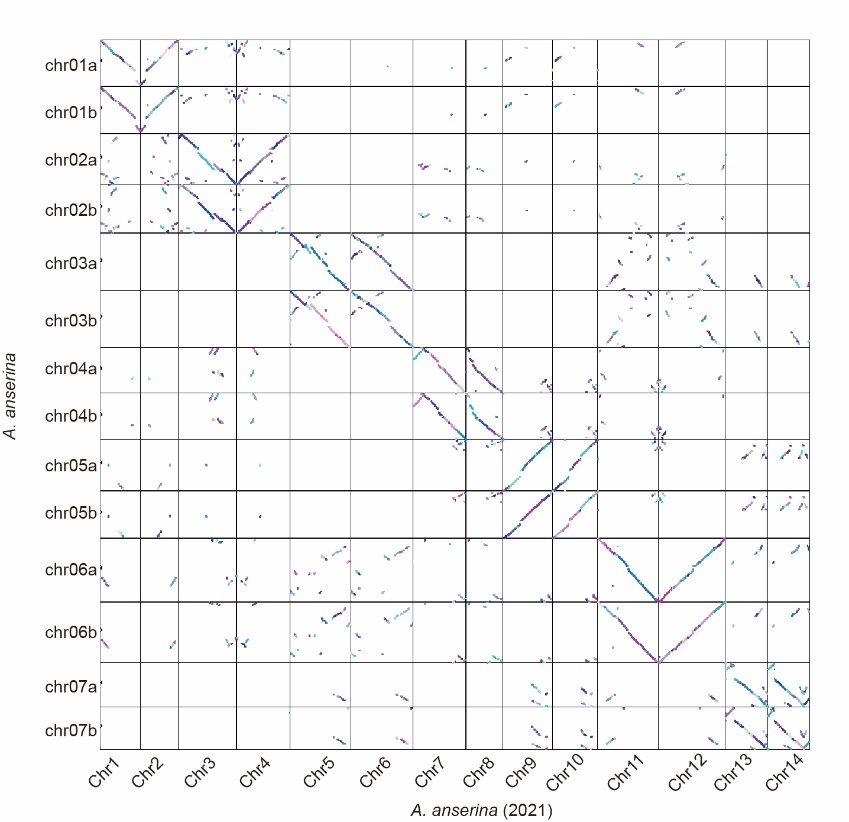


**Supplemental Figure S3** Collinearity analysis of *A. anserina* and *A. anserina* (2010).


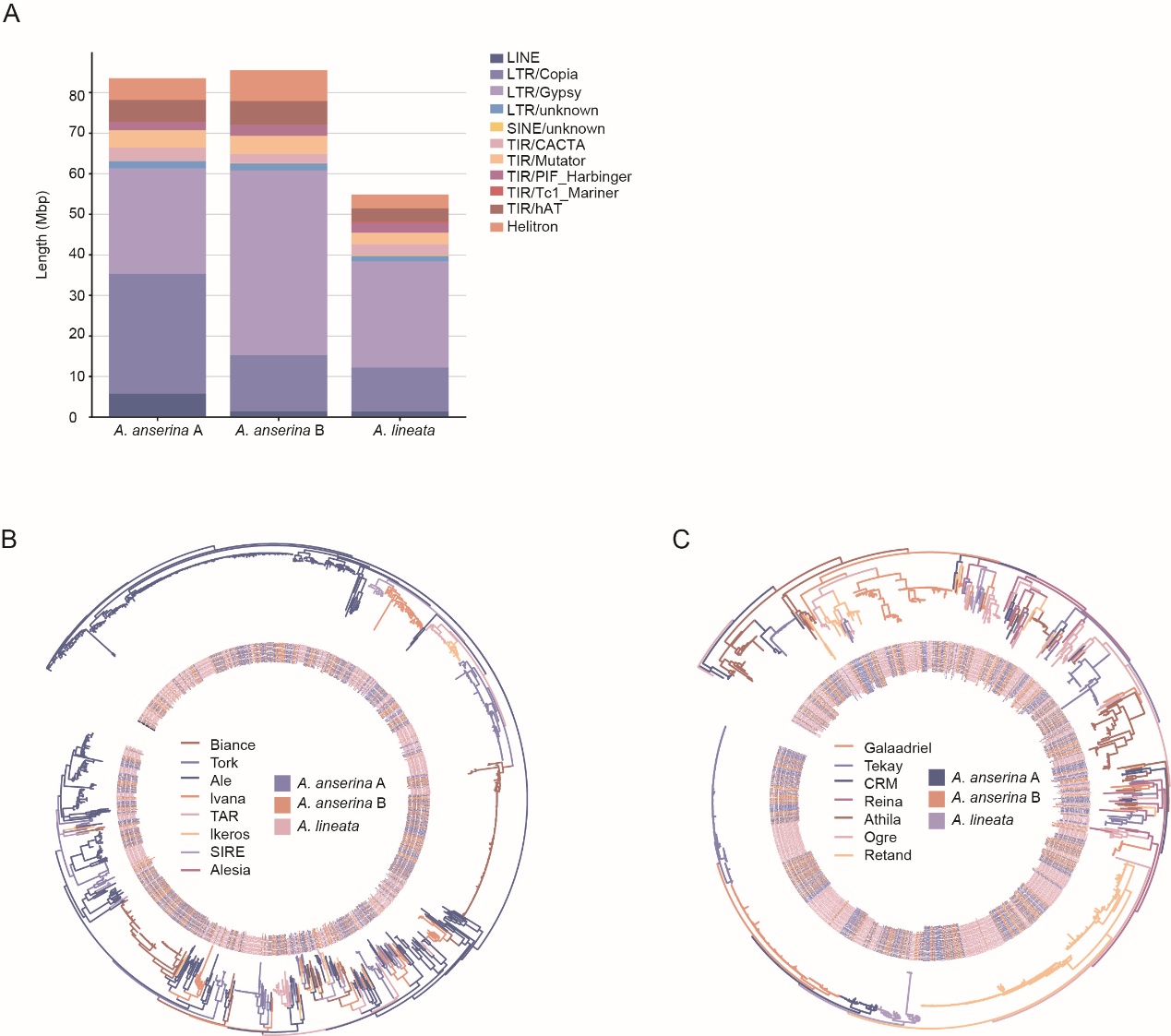


**Supplemental Figure S4** Analysis of transposable element (TE) of *A. anserina* A, *A. anserina* B and *A. lineata.* (A)Sequence size statistics of TE of different types in *A. anserina* A, *A. anserina* B and *A. lineata.* (B) Phylogenetic tree of Ty1/Copia. (C) Phylogenetic tree of Ty3/Gypsy.


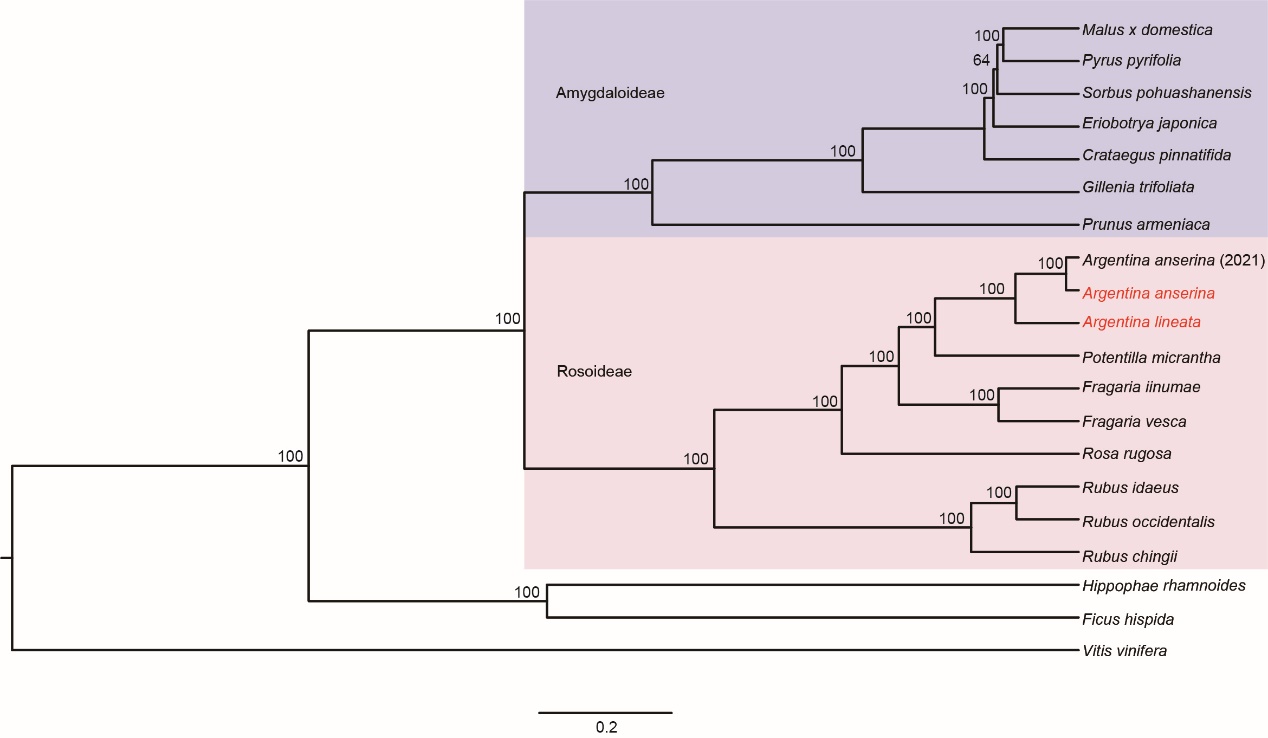


**Supplemental Figure S5** Phylogenetic tree of 19 species. Species in red font are data from this study and numbers on the phylogenetic tree are bootstrap values.


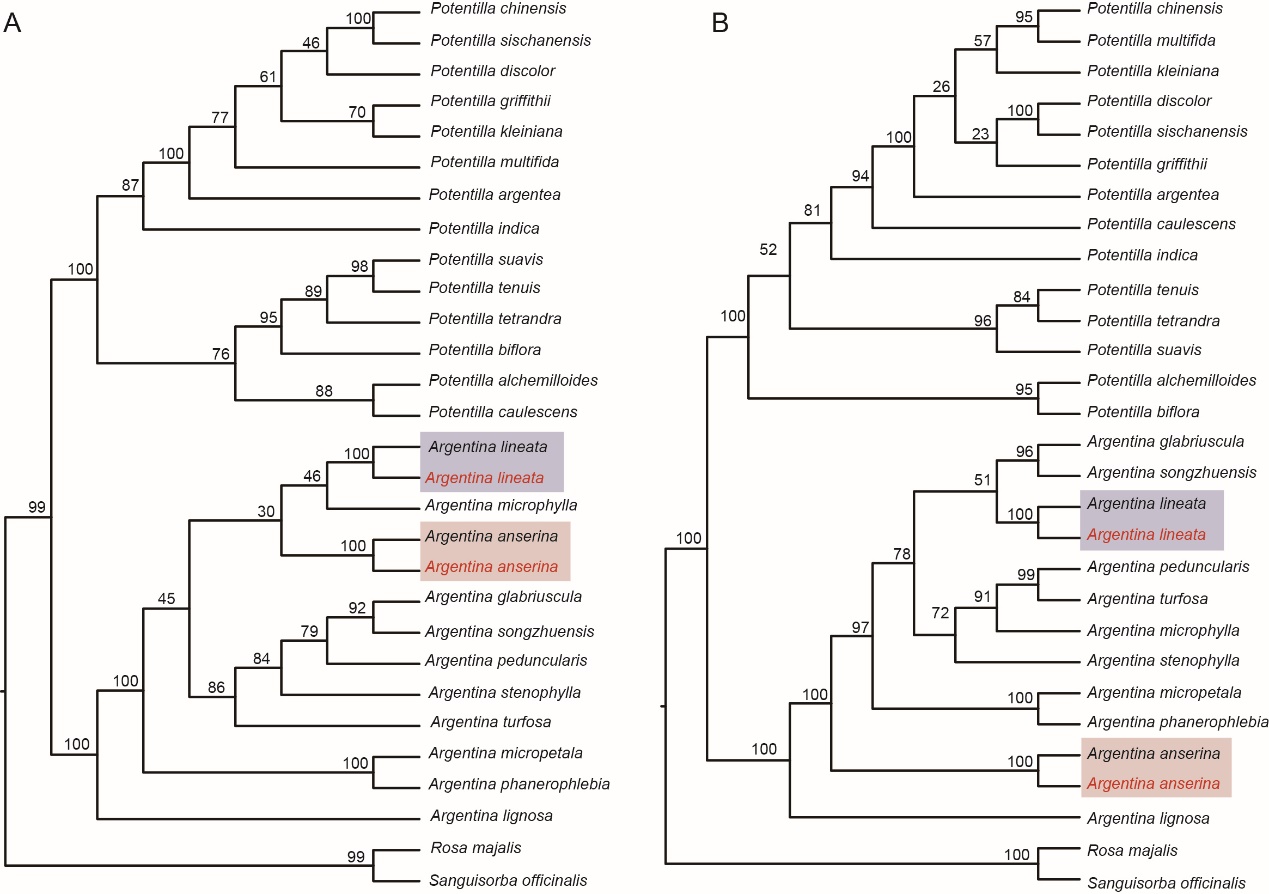


**Supplemental Figure S6** Phylogenetic tree of 25 Potentilleae species. Species in red font are data from this study and numbers on the phylogenetic tree are ML bootstrap values. (A) Phylogenetic tree constructed by ITS and ETS. (B) Phylogenetic tree constructed by *trnL-trnF* and *trnG-trnS*.


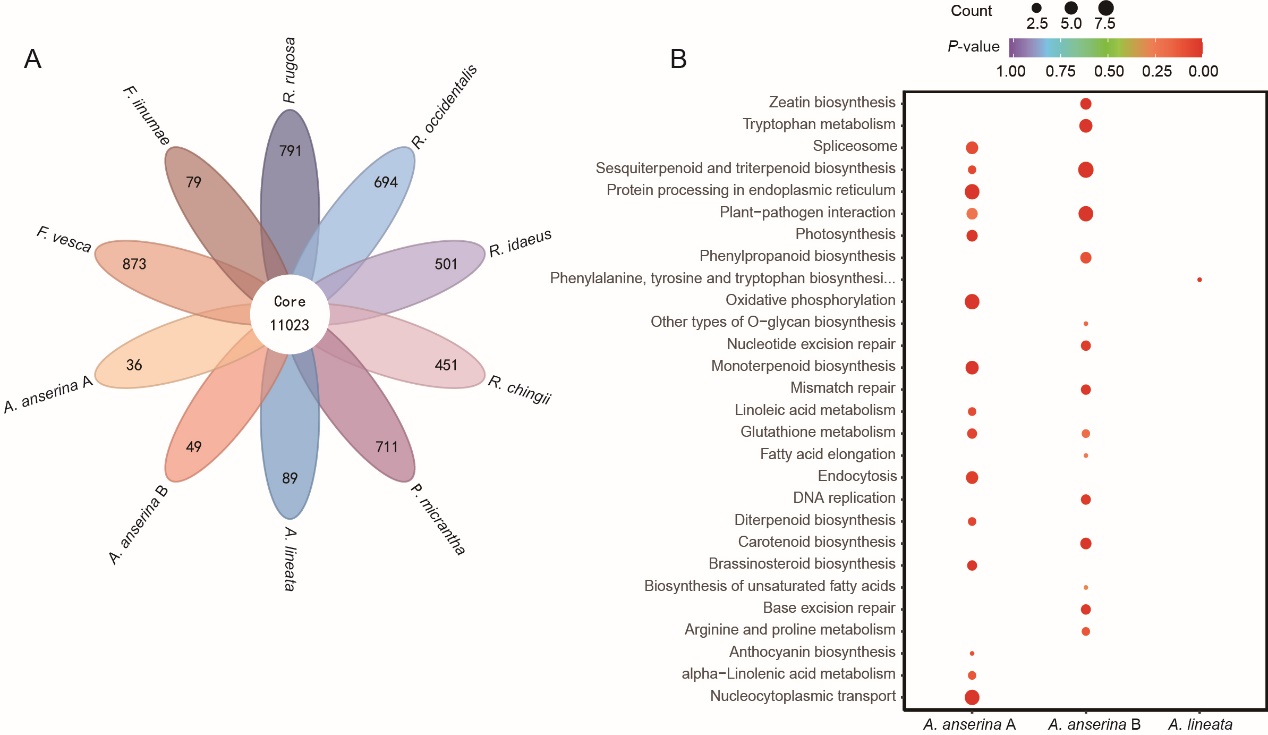


**Supplemental Figure S7** Gene family analysis. (A) Gene families clustering flower plot of 7 other species of the *A. lineata*, *A. anserina*, and Rosoideae subfamilies. The center of the flower represents the common gene families, and the petal represents the species-specific gene families. (B) KEGG enrichment analysis of contraction genes.


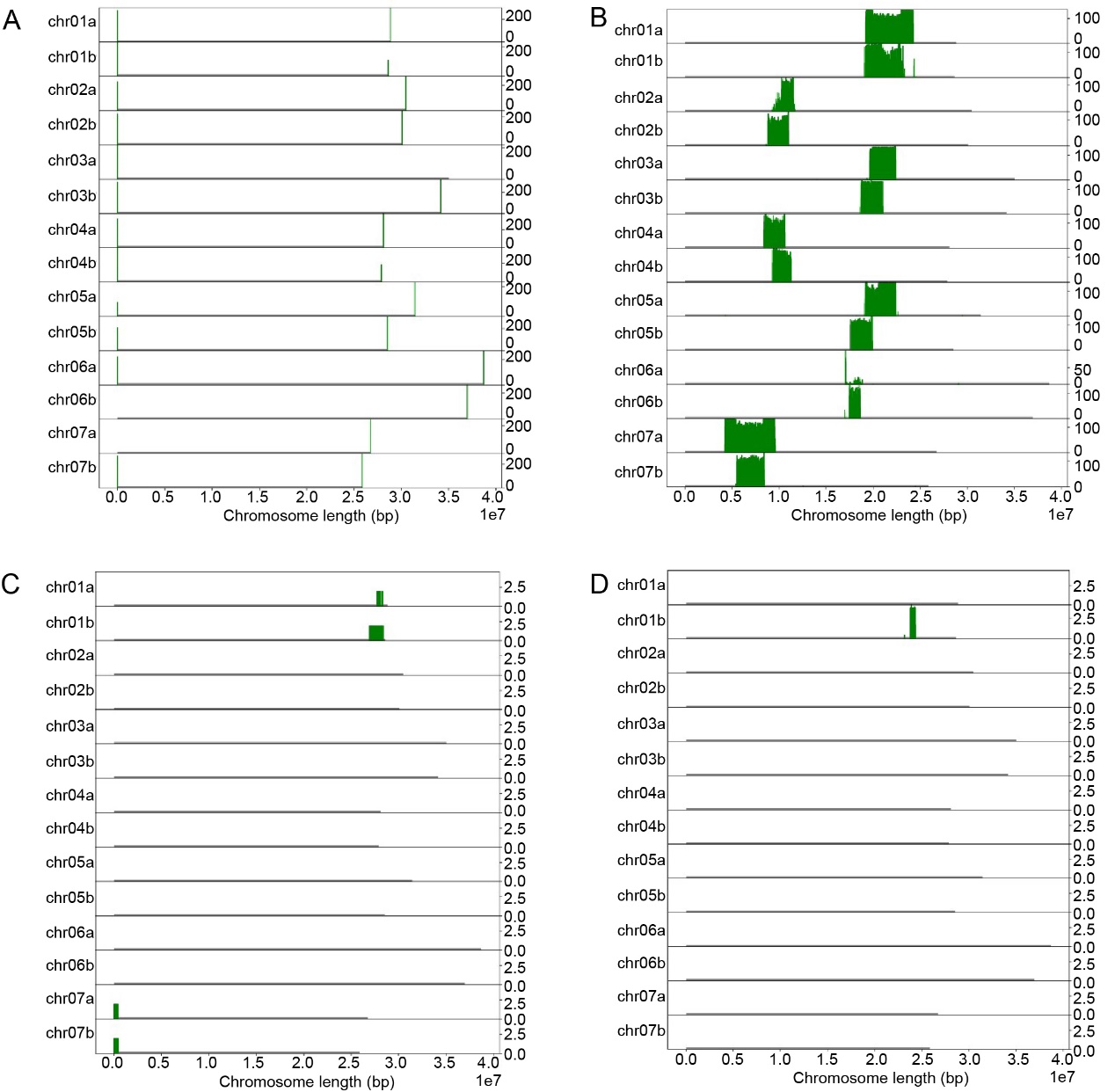


**Supplemental Figure S8** Distribution of the characteristic sequences of *A. anserina* on chromosomes. (A) Telomere characteristic sequences. (B) Highly tandemly repeated sequences. (C) 18-5.8-28S rDNA. (D) 5S rDNA.


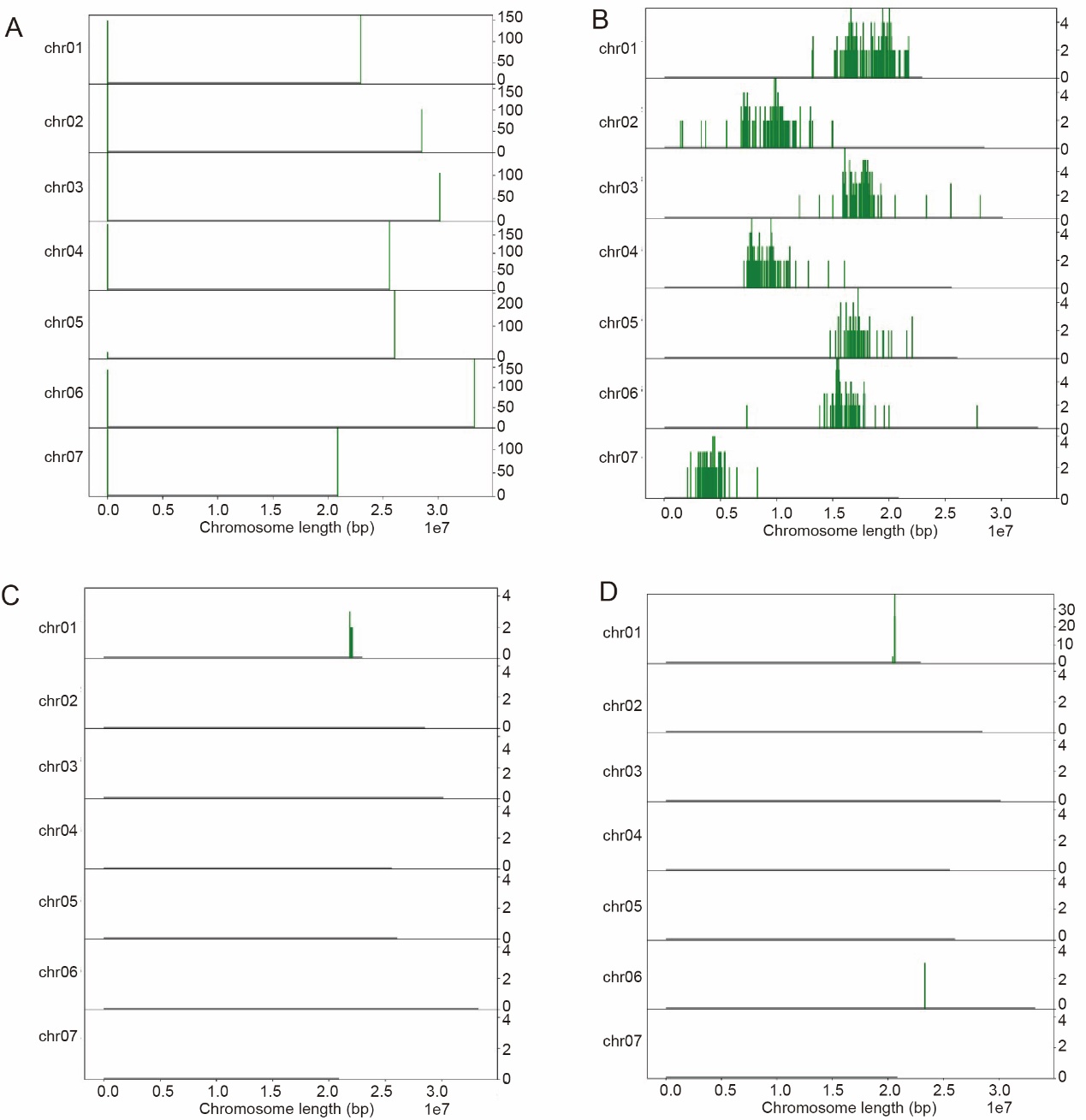


**Supplemental Figure S9** Distribution of the characteristic sequences of *A. lineata* on chromosomes. (A) Telomere characteristic sequences. (B) Highly tandemly repeated sequences. (C) 18-5.8-28S rDNA. (D) 5S rDNA.

**References**

Buchfink B, Xie C, Huson DH. Fast and sensitive protein alignment using DIAMOND. Nat. Methods. 2015;12(1):59-60. <https://doi.org/10.1038/nmeth.3176>.

Cantarel BL, Korf I, Robb SM, Parra G, Ross E, Moore B, Holt C, Alvarado AS, Yandell M. MAKER: an easy-to-use annotation pipeline designed for emerging model organism genomes. Genome research. 2008;18(1):188-196. <https://doi.org/10.1101/gr.6743907>.

Capella-Gutiérrez S, Silla-Martínez JM, Gabaldón T. trimAl: a tool for automated alignment trimming in large-scale phylogenetic analyses. Bioinformatics. 2009;25(15):1972-1973. <https://doi.org/10.1093/bioinformatics/btp348>.

Cheng HY, Concepcion GT, Feng XW, Zhang HW, Li H. Haplotype-resolved de novo assembly using phased assembly graphs with hifiasm. Nat. Methods. 2021;18(2):170-175. <https://doi.org/10.1038/s41592-020-01056-5>.

Chengjie C, Hao C, Yi Z, Hannah R T, Margaret H F, Yehua H, Rui X. TBtools: An Integrative Toolkit Developed for Interactive Analyses of Big Biological Data. Mol Plant. 2020;13(8)<https://doi.org/10.1016/j.molp.2020.06.009>.

Dudchenko O, Batra SS, Omer AD, Nyquist SK, Hoeger M, Durand NC, Shamim MS, Machol I, Lander ES, Aiden AP. De novo assembly of the Aedes aegypti genome using Hi-C yields chromosome-length scaffolds. Science. 2017;356(6333):92-95. <https://doi.org/10.1126/science.aal3327>.

Durand NC, Shamim MS, Machol I, Rao SS, Huntley MH, Lander ES, Aiden EL. Juicer provides a one-click system for analyzing loop-resolution Hi-C experiments. Cell Syst. 2016;3(1):95-98. <https://doi.org/10.1016/j.cels.2016.07.002>.

Emms DM, Kelly S. OrthoFinder: phylogenetic orthology inference for comparative genomics. Genome Biol. 2019;20:1-14. <https://doi.org/10.1186/s13059-019-1832-y>.

Feng T, Moore MJ, Yan MH, Sun YX, Zhang HJ, Meng AP, Li XD, Jian SG, Li JQ, Wang HC. Phylogenetic study of the tribe Potentilleae (Rosaceae), with further insight into the disintegration of Sibbaldia. J. Syst. Evol. 2017;55(3):177-191. <https://doi.org/10.1111/jse.12243>.

Fu LM, Niu BF, Zhu ZW, Wu ST, Li WZ. CD-HIT: accelerated for clustering the next-generation sequencing data. Bioinformatics. 2012;28(23):3150-3152. <https://doi.org/10.1093/bioinformatics/bts565>.

Goel M, Sun HQ, Jiao WB, Schneeberger K. SyRI: identification of syntenic and rearranged regions from whole-genome assemblies. bioRxiv. 2019;1<https://doi.org/10.1093/bioinformatics/bts565>.

Grabherr MG, Haas BJ, Yassour M, Levin JZ, Thompson DA, Amit I, Adiconis X, Fan L, Raychowdhury R, Zeng QD. Full-length transcriptome assembly from RNA-Seq data without a reference genome. Nat. Biotechnol. 2011;29(7):644-652. <https://doi.org/10.1038/nbt.1883>.

Haas BJ, Delcher AL, Mount SM, Wortman JR, Smith Jr RK, Hannick LI, Maiti R, Ronning CM, Rusch DB, Town CD. Improving the Arabidopsis genome annotation using maximal transcript alignment assemblies. Nucleic Acids Res. 2003;31(19):5654-5666. <https://doi.org/10.1093/nar/gkg770>.

Haas BJ, Salzberg SL, Zhu W, Pertea M, Allen JE, Orvis J, White O, Buell CR, Wortman JR. Automated eukaryotic gene structure annotation using EVidenceModeler and the Program to Assemble Spliced Alignments. Genome Biol. 2008;9:1-22. <https://doi.org/10.1186/gb-2008-9-1-r7>.

Hu J, Fan JP, Sun ZY, Liu SL. NextPolish: a fast and efficient genome polishing tool for long-read assembly. Bioinformatics. 2020;36(7):2253-2255. <https://doi.org/10.1093/bioinformatics/btz891>.

Huerta-Cepas J, Forslund K, Coelho LP, Szklarczyk D, Jensen LJ, Von Mering C, Bork P. Fast genome-wide functional annotation through orthology assignment by eggNOG-mapper. Mol. Biol. Evol. 2017;34(8):2115-2122. <https://doi.org/10.1093/molbev/msx148>.

Jin JJ, Yu WB, Yang JB, Song Y, DePamphilis CW, Yi TS, Li DZ. GetOrganelle: a fast and versatile toolkit for accurate de novo assembly of organelle genomes. Genome Biol. 2020;21:1-31. <https://doi.org/10.1186/s13059-020-02154-5>.

Jones P, Binns D, Chang H-Y, Fraser M, Li W, McAnulla C, McWilliam H, Maslen J, Mitchell A, Nuka G. InterProScan 5: genome-scale protein function classification. Bioinformatics. 2014;30(9):1236-1240. <https://doi.org/10.1093/bioinformatics/btu031>.

Katoh K, Standley DM. MAFFT multiple sequence alignment software version 7: improvements in performance and usability. Mol. Biol. Evol. 2013;30(4):772-780. <https://doi.org/10.1093/molbev/mst010>.

Kim D, Langmead B, Salzberg SL. HISAT: a fast spliced aligner with low memory requirements. Nat. Methods. 2015;12(4):357-360. <https://doi.org/10.1038/nmeth.3317>.

Kumar S, Suleski M, Craig JM, Kasprowicz AE, Sanderford M, Li M, Stecher G, Hedges SB. TimeTree 5: an expanded resource for species divergence times. Mol. Biol. Evol. 2022;39(8):msac174. <https://doi.org/10.1093/molbev/msac174>.

Langdon QK, Peris D, Kyle B, Hittinger CT. sppIDer: a species identification tool to investigate hybrid genomes with high-throughput sequencing. Mol. Biol. Evol. 2018;35(11):2835-2849. <https://doi.org/10.1093/molbev/msy166>.

Li H. Aligning sequence reads, clone sequences and assembly contigs with BWA-MEM. arXiv preprint arXiv:1303.3997. 2013;<https://doi.org/10.48550/arXiv.1303.3997>.

Li H. Minimap2: pairwise alignment for nucleotide sequences. Bioinformatics. 2018;34(18):3094-3100. <https://doi.org/10.1093/bioinformatics/bty191>.

Lowe TM, Eddy SR. tRNAscan-SE: a program for improved detection of transfer RNA genes in genomic sequence. Nucleic Acids Res. 1997;25(5):955-964. <https://doi.org/10.1093/nar/25.5.955>.

Mendes FK, Vanderpool D, Fulton B, Hahn MW. CAFE 5 models variation in evolutionary rates among gene families. Bioinformatics. 2020;36(22-23):5516-5518. <https://doi.org/10.1093/bioinformatics/btaa1022>.

Minh BQ, Schmidt HA, Chernomor O, Schrempf D, Woodhams MD, Von Haeseler A, Lanfear R. IQ-TREE 2: new models and efficient methods for phylogenetic inference in the genomic era. Mol. Biol. Evol. 2020;37(5):1530-1534. <https://doi.org/10.1093/molbev/msaa015>.

Nawrocki EP, Burge SW, Bateman A, Daub J, Eberhardt RY, Eddy SR, Floden EW, Gardner PP, Jones TA, Tate J. Rfam 12.0: updates to the RNA families database. Nucleic Acids Res. 2015;43(D1):D130-D137. <https://doi.org/10.1093/nar/gku1063>.

Ou S, Su W, Liao Y, Chougule K, Agda JR, Hellinga AJ, Lugo CSB, Elliott TA, Ware D, Peterson T. Benchmarking transposable element annotation methods for creation of a streamlined, comprehensive pipeline. Genome Biol. 2019;20:1-18. <https://doi.org/10.1186/s13059-019-1905-y>.

Pertea M, Pertea GM, Antonescu CM, Chang T-C, Mendell JT, Salzberg SL. StringTie enables improved reconstruction of a transcriptome from RNA-seq reads. Nat. Biotechnol. 2015;33(3):290-295. <https://doi.org/10.1038/nbt.3122>.

Pryszcz LP, Gabaldón T. Redundans: an assembly pipeline for highly heterozygous genomes. Nucleic Acids Res. 2016;44(12):e113-e113. <https://doi.org/10.1093/nar/gkw294>.

Quinlan AR, Hall IM. BEDTools: a flexible suite of utilities for comparing genomic features. Bioinformatics. 2010;26(6):841-842. <https://doi.org/10.1093/bioinformatics/btq033>.

Robinson JT, Turner D, Durand NC, Thorvaldsdóttir H, Mesirov JP, Aiden EL. Juicebox. js provides a cloud-based visualization system for Hi-C data. Cell Syst. 2018;6(2):256-258. e251. <https://doi.org/10.1016/j.cels.2018.01.001>.

Simão FA, Waterhouse RM, Ioannidis P, Kriventseva EV, Zdobnov EM. BUSCO: assessing genome assembly and annotation completeness with single-copy orthologs. Bioinformatics. 2015;31(19):3210-3212. <https://doi.org/10.1093/bioinformatics/btv351>.

Stamatakis A. RAxML version 8: a tool for phylogenetic analysis and post-analysis of large phylogenies. Bioinformatics. 2014;30(9):1312-1313. <https://doi.org/10.1093/bioinformatics/btu033>.

Stanke M, Diekhans M, Baertsch R, Haussler D. Using native and syntenically mapped cDNA alignments to improve de novo gene finding. Bioinformatics. 2008;24(5):637-644. <https://doi.org/10.1093/bioinformatics/btn013>.

Wang YP, Tang HB, DeBarry JD, Tan X, Li JP, Wang XY, Lee T-h, Jin HZ, Marler B, Guo H. MCScanX: a toolkit for detection and evolutionary analysis of gene synteny and collinearity. Nucleic Acids Res. 2012;40(7):e49-e49. <https://doi.org/10.1093/nar/gkr1293>.

Wu T, Hu E, Xu S, Chen M, Guo P, Dai Z, Feng T, Zhou L, Tang W, Zhan L, Fu X, Liu S, Bo X, Yu G. clusterProfiler 4.0: A universal enrichment tool for interpreting omics data. Innovation (Camb). 2021;2(3):100141. <https://doi.org/10.1016/j.xinn.2021.100141>.

Xu GC, Xu TJ, Zhu R, Zhang Y, Li SQ, Wang HW, Li JT. LR_Gapcloser: a tiling path-based gap closer that uses long reads to complete genome assembly. GigaScience. 2019;8(1):giy157. <https://doi.org/10.1093/gigascience/giy157>.

Yan HD, Bombarely A, Li S. DeepTE: a computational method for de novo classification of transposons with convolutional neural network. Bioinformatics. 2020;36(15):4269-4275. <https://doi.org/10.1093/bioinformatics/btaa519>.

Yang ZH. PAML 4: phylogenetic analysis by maximum likelihood. Mol. Biol. Evol. 2007;24(8):1586-1591. <https://doi.org/10.1093/molbev/msm088>.

Zhang R-G, Shang H-Y, Zhou M-J, Shu H, Jia K-H, Ma Y-P. Robust identification of orthologous synteny with the Orthology Index and its applications in reconstructing the evolutionary history of plant genomes. bioRxiv. 2024:2024.2008. 2022.609065. <https://doi.org/10.1101/2024.08.22.609065>.

Zhang RG, Li GY, Wang XL, Dainat J, Wang ZX, Ou SJ, Ma YP. TEsorter: an accurate and fast method to classify LTR-retrotransposons in plant genomes. Hortic. Res. 2022;9:uhac017. <https://doi.org/10.1093/hr/uhac017>.

Zhang Z, Xiao JF, Wu JY, Zhang HY, Liu GM, Wang XM, Dai L. ParaAT: a parallel tool for constructing multiple protein-coding DNA alignments. Biochem. Biophys. Res. Commun. 2012;419(4):779-781. <https://doi.org/10.1016/j.bbrc.2012.02.101>.
